# A novel splice-site mutation of *TULP1* underlies severe early-onset retinitis pigmentosa in a consanguineous Israeli Muslim Arab family

**Published:** 2008-04-21

**Authors:** Anan H. Abbasi, Hanna J. Garzozi, Tamar Ben-Yosef

**Affiliations:** 1 Department of Genetics and The Rappaport Family Institute for Research in the Medical Sciences, Rappaport Faculty of Medicine, Technion-Israel Institute of Technology, Haifa, Israel; 2 Department of Ophthalmology, Bnai Zion Medical Center, Haifa, Israel

## Abstract

**Purpose:**

To investigate the genetic basis for autosomal recessive severe early-onset retinitis pigmentosa (RP) in a consanguineous Israeli Muslim Arab family.

**Methods:**

Haplotype analysis for all known genes underlying autosomal recessive RP was performed. Mutation screening of the underlying gene was done by direct sequencing. An in vitro splicing assay was used to evaluate the effect of the identified mutation on splicing.

**Results:**

Haplotype analysis indicated linkage to the *Tubby-like protein 1* (*TULP)1* gene. Direct sequencing revealed a homozygous single base insertion, c.1495+2_1495+3insT, located in the conserved donor splice-site of intron 14. This mutation co-segregated with the disease, and was not detected in 114 unrelated Israeli Muslim Arab controls. We used an in vitro splicing assay to demonstrate that this mutation leads to incorrect splicing.

**Conclusions:**

To date, 22 distinct pathogenic mutations of *TULP1* have been reported in patients with early-onset RP or Leber congenital amaurosis. Here we report a novel splice-site mutation of *TULP1*, c.1495+2_1495+3insT, underlying autosomal recessive early-onset RP in a consanguineous Israeli Muslim Arab family. This report expands the spectrum of pathogenic mutations of the *TULP1* gene.

## Introduction

Inherited retinal degenerations are a clinically and genetically heterogeneous group of diseases. Many of them cause visual loss due to premature death of rod and cone photoreceptors. Leber congenital amaurosis (LCA) is the most severe form of hereditary retinal degeneration and the most common genetic cause of congenital visual impairment in infants and young children. In these children both rods and cones have degenerated or are nonfunctional at birth, or are lost within the first years of life, and thus electroretinographic (ERG) responses are extinguished or severely reduced [1-3]. Retinitis pigmentosa (RP) is a relatively less severe form of retinal degeneration. In most forms of RP, rod degeneration exceeds cone degeneration; thus the first clinical symptom is usually night blindness, followed by gradual restriction of the visual field. As the disease progresses, both rods and cones degenerate. Additional ophthalmologic findings include characteristic pigmentation of the midperipheral retina, attenuation of the retinal arterioles, and pale appearance of the optic disc [4]. The diagnostic boundary separating severe early-onset RP from LCA is not distinct, and in some cases both diagnoses may apply [4,5].

Both LCA and RP are genetically heterogeneous disorders. In most patients, the disease is limited to the eye (nonsyndromic RP/LCA), with no extraocular manifestations. Nonsyndromic RP can be inherited as autosomal recessive, autosomal dominant, or X-linked. Mitochondrial and digenic patterns of inheritance have also been described [5]. Over 45 genes and loci have been implicated in nonsyndromic RP, of which at least 29 are associated with an autosomal recessive mode of inheritance. LCA is most frequently inherited as autosomal recessive, although autosomal dominant forms have also been described [1]. Fifteen genes and loci have been implicated in nonsyndromic LCA (Retnet; Retinal Information Network).

One of the genes underlying both autosomal recessive RP and LCA is *TULP1*, encoding tubby-like protein 1. TULP1 is a member of the tubby-like family of proteins, including TUB and TULP1–3. These proteins are defined by the highly conserved “tubby domain” located in their COOH-terminal part. TULP proteins are found in multicellular organisms, and all four family members are conserved among vertebrate genomes. TULPs are localized primarily to the nervous system, and all of them are expressed in the retina. Although the exact function of TULP proteins is not well understood, these proteins appear to play an important role in neuronal development and function [6]. Two of the TULP family members have been linked to retinal degeneration. A naturally occurring null mutation in the *tubby* gene causes photoreceptor and cochlear degeneration and adult-onset obesity in mice [7,8]. Mutations of *TULP1* cause retinal degeneration in both humans and mice [9-12]. Recent studies indicate that TULP1 is involved in the vesicular trafficking of photoreceptor proteins, both in the nerve terminal during synaptic transmission, and at the inner segment during protein translocation to the outer segment [13-15].

To date, 22 distinct pathogenic mutations of *TULP1* have been reported in patients with LCA or RP (Table 1) [2,16-21]. Here we report a novel splice-site mutation of *TULP1*, c.1495+2_1495+3insT, underlying severe early-onset RP in a consanguineous Israeli Muslim Arab family.

## Methods

### Patients

Nine members (six males and three females) of a Muslim Arab consanguineous family from Northern Israel (family TB13) were ascertained for this study. They included three affected individuals (31, 38, and 41 years old), and six unaffected individuals (39, 40, 42, 45, 68, and 70 years old). The study was performed in accordance with the Declaration of Helsinki and written informed consent was obtained from all participants. The research was approved by the local institutional review board at Bnai Zion Medical Center and by the National Helsinki Committee for Genetic Research in Humans. Digital fundus photographs were taken with a Zeiss 450 Plus fundus camera (Carl Zeiss, Inc., Oberkochen, Germany), combined with an OIS WinStation 3200 digital system (Ophthalmic Imaging Systems, Inc., Sacramento, CA). DNA control samples were obtained from 114 Muslim Arab individuals from Northern Israel (both males and females, 20–40 years old) who presented for routine genetic testing and consented for the use of their DNA samples in additional genetic studies.

**Figure 1:**
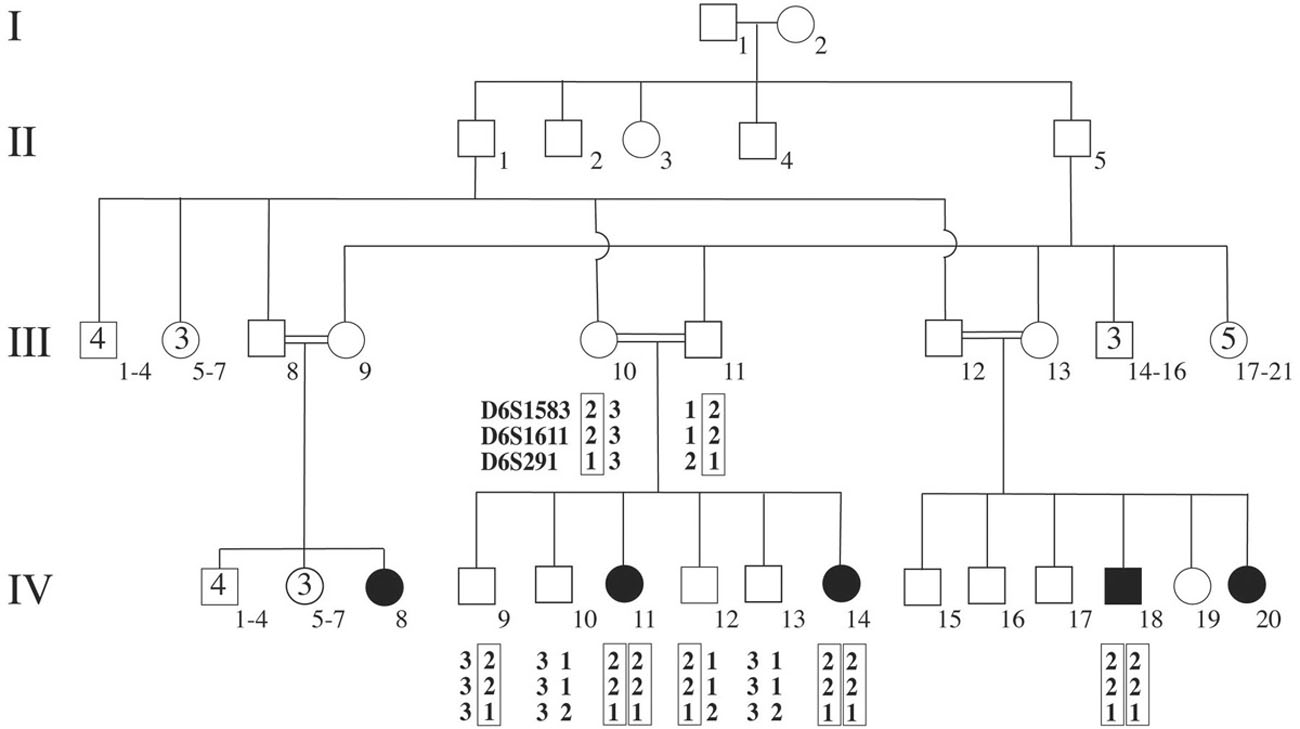
Pedigree and haplotype analysis. Shown is a pedigree of a consanguineous Israeli Muslim Arab family segregating early-onset autosomal recessive retinitis pigmentosa (family TB13). Haplotype analysis performed on this family demonstrated co-segregation of a haplotype of three polymorphic marker alleles, linked to *TULP1* on chromosome 6p21.31, with autosomal recessive retinitis pigmentosa in this family. The mutation-bearing haplotype is marked by a box. Males are represented by squares, females are represented by circles. Affected individuals are marked by black shadings.

### Haplotype analysis

Genomic DNA was extracted from venous blood samples according to a standard protocol [22]. DNA samples were PCR amplified with fluorescent dye-labeled primers flanking microsatellite repeat markers linked to all known autosomal recessive RP and LCA genes and loci (Retnet; Retinal Information Network). The microsatellite repeat markers used to test for linkage to *TULP1* were D6S1583, D6S1611, and D6S291, which span an interval of 2.5 Mb flanking the gene. PCR products were separated by electrophoresis on an ABI 310 Genetic Analyzer (PE Applied Biosystems, Foster City, CA), and genotypes were determined by Genescan and Genotyper software, using a Genescan-400HD-ROX size standard (PE Applied Biosystems).

### Mutation analysis

Specific primers were used to PCR amplify the 15 exons of *TULP1*, including intron-exon boundaries [2]. PCR products were purified with the Wizard SV PCR clean-up system (Promega Corporation, Madison, WI). Mutation screening was performed by direct sequencing with the Big Dye terminator cycle sequencing kit on an ABI 3130xl Genetic Analyzer (PE Applied Biosystems). To screen control DNA samples for the c.1495+2_1495+3insT mutation, we created a mismatch-primer restriction-based assay. Briefly, DNA samples were subjected to amplification in a 25 μl reaction volume with the following primers: F-CTT CTC CGG TCA GTC CTG AG and R- GTG GAG AAG AGC CAG ACC TGG GCC CTC AGG GAC TC. The reverse primer includes one mismatched base (underlined), which, in combination with the mutant allele, but not with the wild-type (WT) allele, creates a DrdI restriction site (GAC NNN NNN GTC). Twenty μl of the PCR product were digested with 3 U DrdI and 1X of the recommended buffer (New England Biolabs, Beverly, MA) for several hours at 37 °C. The entire digestion volume (30 μl) was visualized by electrophoresis on a 5% NuSieve 3:1 agarose gel (Cambrex Bio Science, Rockland, ME) in 1XTBE buffer. Expected band sizes were 286 bp for the WT allele and 251 and 35 bp for the mutant allele.

**Table 1:** Currently known mutations of the *TULP1* gene

Exon/Intron	Base change	Amino acid substitution	Phenotype	Notes	Reference
Intron 2	c.99+1G>A	Aberrant splicing	LCA/Early onset RP	Found in compound heterozygosity with p.E402X and with p.I459K	[2,11]
Intron 4	c.350-2delAGA, (IVS4-2delAGA)	Aberrant splicing	Early onset RP		[21]
Exon 5	c.394del24	p.E120-D127del	RP	Found in heterozygosity; second mutant allele not identified	[18]
Intron 7	c.718+2T>C	Aberrant splicing	Juvenile RP		[16]
Exon 10	c.937delC	p.Q301fsX8	Early onset RP		[21]
Intron 10	c.999+5G>C	Aberrant splicing	Juvenile RP		[16]
Exon 11	c.1102G>T	p.G368W	LCA		[2]
Exon 11	c.1145T>C	p.F382S	Early onset RP		[19]
Exon 12	c.1198C>T	p.R400W	LCA		[2]
Exon 12	c.1204G>T	p.E402X	LCA	Found in compound heterozygosity with c.99+1G>A	[2]
Intron 12	c.1224+4A>G, (IVS12+4A>G)	Aberrant splicing	RP	Found in heterozygosity; second mutant allele not identified	[18]
Exon 13	c.1259G>C	p.R420P	Early onset RP	Found in compound heterozygosity with p.F491L	[11]
Exon 14	c.1376 T>A	p.I459K	Early onset RP	Found in compound heterozygosity with c.99+1G>A	[11]
Exon 14	c.1381C>G	p.L461V	Juvenile RP		[16]
Exon 14	c.1472 T>C	p.F491L	Early onset RP	Found in compound heterozygosity with p.R420P	[11]
Exon 14	c.1444C>T	p.R482W	Early onset RP	Found in compound heterozygosity with p.L504fsX140	[17]
Exon 14	c.1502G>A	p.K489R	RP		[18]
Exon 14	c.1522 G>A	p.A496T	RP	Found in heterozygosity; second mutant allele not identified	[18]
Intron 14	c.1495+1G>A, (IVS14+1G>A)	Aberrant splicing	Early onset RP		[9,24]
Intron 14	c.1495+2_1495+3insT	Aberrant splicing	Early onset RP		Current report
Intron 14	c.1496-6C>A, (IVS14-6C>A)	Aberrant splicing	RP		[18]
Exon 15	c.1511-1521del, TGCAGTTCGGC	p.L504fsX140	Early onset RP	Found in compound heterozygosity with p.R482W	[17]
Exon 15	c.1593-1594 dup, TTCGCC	p.FA531-532dup	LCA/Early onset RP		[20]

Abbreviations: RP represents retinitis pigmentosa, LCA represents Leber congenital amaurosis.

### Splice-site score predictions

The genomic sequence environment of the mutation was analyzed for 5′ and 3′ splice-sites using Splice Site Prediction by Neural Network, Splice Site Finder, and MaxEntScan.

### In vitro splicing assay

To create wt and mutant minigene constructs, we PCR-amplified DNA fragments harboring *TULP1* exons 12, 13, 14, and 15, each flanked by 79–183 bp of intronic sequences, from genomic DNA derived from a patient homozygote for the c.1495+2_1495+3insT mutation or from a normal control. The c.1495+1G>A mutation was generated by site-directed mutagenesis, using the QuikChange Site-Directed Mutagenesis Kit (Stratagene, La Jolla, CA). The four fragments were inserted in tandem into the pCMV-Script mammalian expression vector (Stratagene). Y79 retinoblastoma cells were obtained from the American Type Culture Collection and cultured in RPMI 1640 medium containing L-glutamine and sodium bicarbonate (Sigma-Aldrich, St Louis, MO), and supplemented with 10 mM HEPES, 1 mM sodium pyruvate, and 20% fetal bovine serum (FBS; Biologic Industries, Beit Haemek, Israel). Cells were maintained at 37 °C and 5% CO_2_. Constructs were transfected into Y79 cells by electroporation. 20×10^6^ cells were electroporated with 15 μg of DNA at room temperature in 0.3 ml of RPMI 1640 containing 20% FBS, using an ECM 830 square wave electroporator (BTX, a subdivision of Genetronics, San Diego, CA) at a setting of 300 V, 10 ms and in LV mode. Twenty-four hours following transfection, total RNA was extracted from cells with TRI reagent (Sigma), and treated with RQ1 RNase-free DNase (Promega). Reverse transcription was performed with 1 μg of total RNA in a 20 μl reaction volume using 200 U of M-MLV Reverse Transcriptase and 100 ng of random primers (Stratagene). Two μl of cDNA were subjected to PCR amplification with the T3 vector-derived forward primer (GCG CGA AAT TAA CCC TCA CTA AAG) and a *TULP1*-specific reverse primer (ACC GGT AGT CTA GGG TGA AG). Cycling conditions were 95 °C for 5 min, followed by 35 cycles of 95 °C for 30 s, 55 °C for 30 s, and 72 °C for 30 s, and a final step of 72 °C for 10 min.

## Results

### Clinical findings

**Figure 2:**
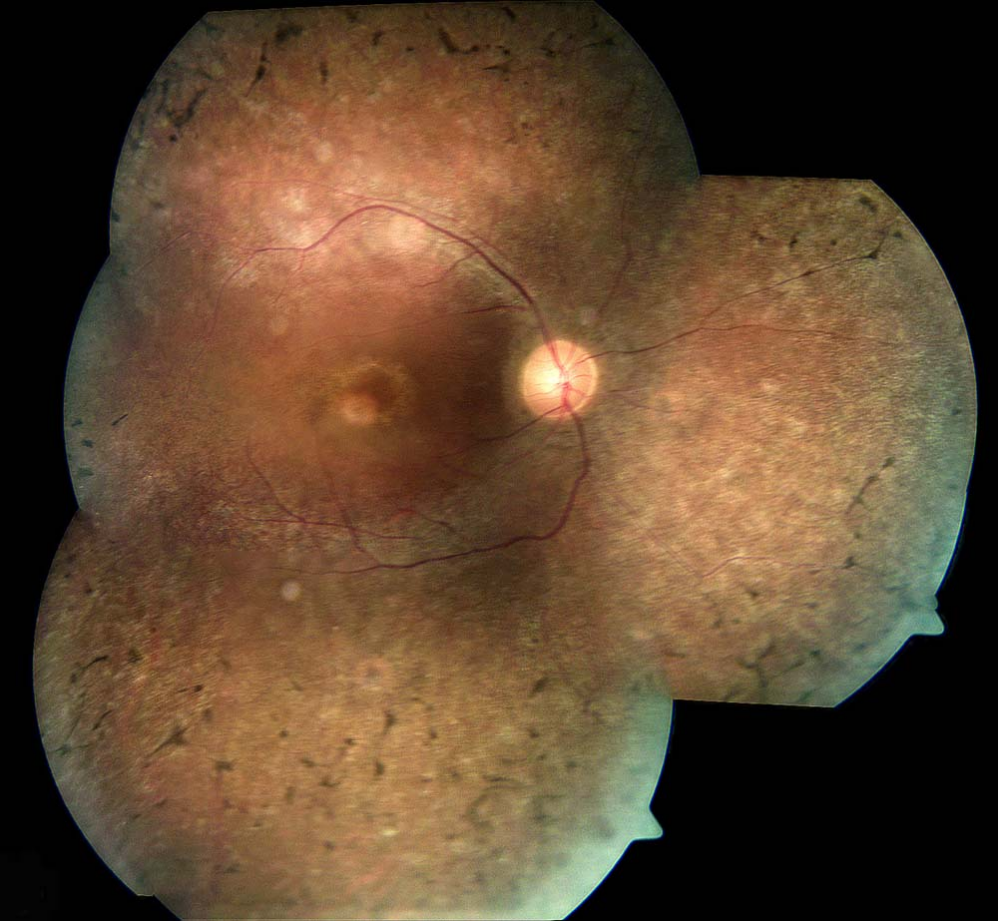
Fundus photographs of individual IV-18 (right eye). The photographs show vascular attenuation, peripheral bone spicule pigmentation, accompanied by salt and pepper-like changes, and signs of maculopathy, including edema and a yellow perifoveal ring.

The family included five affected individuals, four of whom live in Israel. In total, nine family members participated in the study (three affected and six unaffected; Figure 1). All affected family members, who are currently over 30 years old, had severe retinal dystrophy since early childhood. Symptoms included low visual acuity, night blindness, and congenital nystagmus. The most detailed early clinical records were available for individual IV-18. He was born with congenital cataract on his left eye, which was surgically removed at the age of one year, with no secondary implantation of an intraocular lens. At the age of eight years he had reduced visual acuity (right eye: 6/30; left eye: 20 cm finger counting). Funduscopic examination at the time revealed vascular attenuation. Both scotopic and photopic ERGs were extinct. Visual evoked potentials (VEP) from both eyes were reduced, although typical waves with normal latency could be detected. At the age of 23 years, his visual acuity deteriorated (right eye: 1/60; left eye: light perception). Funduscopic findings at this age included retinal vascular attenuation and bone spicule-type pigmentation. No consistent VEP responses were obtained. The reduced VEP responses, in addition to the extinct ERG responses, indicate impairment of both peripheral and macular function. This conclusion is supported by funduscopic findings at the age of 31 years, demonstrating both peripheral and macular abnormalities. These include vascular attenuation, peripheral bone spicule-type pigment deposits, accompanied by salt and pepper-like changes, and signs of maculopathy (Figure 2). Current visual acuity is 30 cm finger counting (right eye), and no light perception (left eye).

### Haplotype analysis

Family TB13 is consanguineous, and retinal degeneration segregates in an autosomal recessive mode. Since signs of retinal dysfunction presented at birth or in very early childhood, we performed haplotype analysis with markers linked to all known genes and loci linked to both autosomal recessive RP and LCA (Retnet; Retinal Information Network). A haplotype of three polymorphic marker alleles (D6S1583, D6S1611, D6S291) linked to *TULP1* on chromosome 6p21.31, co-segregated with retinal degeneration in this family (Figure 1).

### Mutation analysis

**Figure 3:**
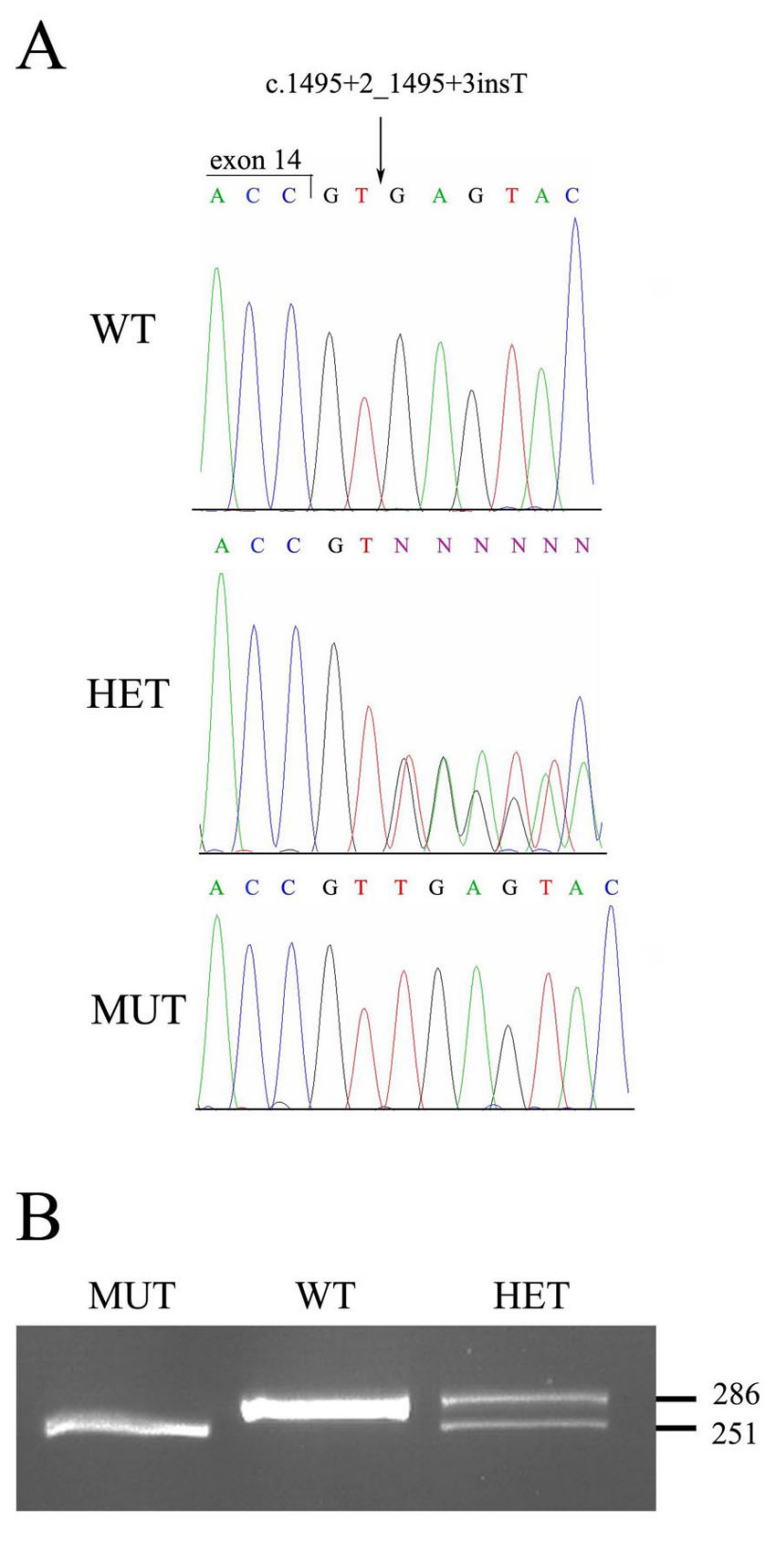
The c.1495+2_1495+3insT mutation. **A**: Shown are nucleotide sequence traces of the boundary between *TULP1* exon and intron14 in a non-carrier individual (wt), an individual heterozygote for the c.1495+2_1495+3insT mutation (het), and an affected individual homozygote for the mutant allele (mut). The exon-intron boundary is marked. **B**: A mismatch-primer restriction–based assay used to screen control DNA samples for the c.1495+2_1495+3insT mutation. The reverse PCR primer included one mismatched base, which, in combination with the mutant allele, but not with the WT allele, created a DrdI restriction site. A 286 bp PCR product derived from the WT allele was not digested by DrdI, while in the presence of the MUT two restriction fragments of 251 and 35 bp were obtained. Digestion of a PCR product derived from a heterozygote for the mutation (HET) yielded three fragments (286, 251, and 35 bp). The 35 bp fragment is not visible.

To detect the pathogenic mutation in *TULP1*, we determined the sequence of each of the 15 exons, including exon-intron boundaries, in one of the affected individuals from family TB13 (Figure 1, individual IV-11). We identified a homozygous T nucleotide insertion between positions +2 and +3 of intron 14, within the conserved donor splice-site (c.1495+2_1495+3insT; see Figure 3A). Analysis of all available family members revealed that the mutation co-segregates with retinal degeneration in this family. Affected individuals are homozygotes for the mutation, while their parents are heterozygotes. Unaffected siblings are either heterozygotes or homozygotes for the WT allele. Heterozygote carriers of the mutation were asymptomatic.

To screen control DNA samples for c.1495+2_1495+3insT, we created a mismatch-primer restriction–based assay (Figure 3B). We found c.1495+2_1495+3insT was not detected in 228 chromosomes derived from unrelated Israeli Muslim Arab control subjects from Northern Israel, indicating that it is not a common polymorphism in this population.

### In vitro splicing assay

According to splice-site consensus sequence in mammals, a purine is located at position +3 of the donor site [23]. Indeed, in the wt *TULP1* allele a G is located at position +3 of intron 14 (UCSC Genome Bioinformatics and Figure 3A). However, in the mutant allele the nucleotide at position +3 is a pyrimidine (T; Figure 3A). To predict the effect of this insertion on splicing, we performed *in silico* analysis of the sequence using three different web-based tools (Splice Site Prediction by Neural Network, Splice Site Finder, and MaxEntScan). All three algorithms predicted that the c.1495+2_1495+3insT mutation would lead to elimination of intron 14 donor site.

**Figure 4:**
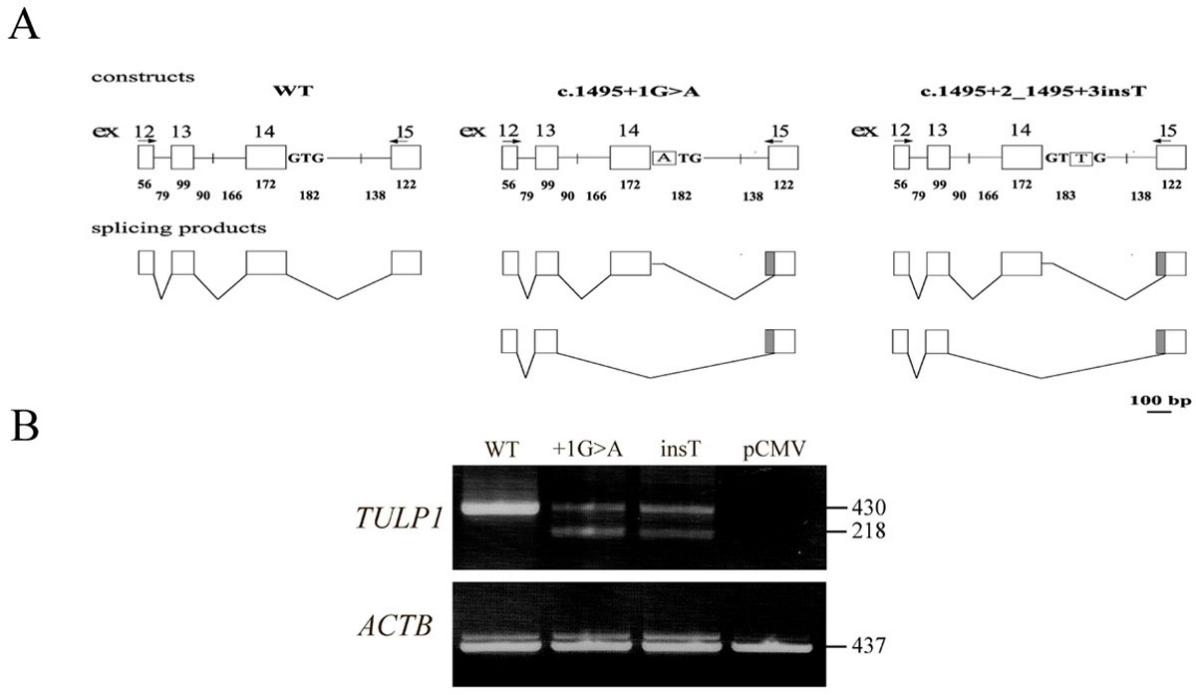
Minigene constructs and products obtained in the in vitro splicing assay. **A**: Shown is a schematic representation of the constructs, which include *TULP1* exons 12 to 15 (represented by boxes), flanked by 79–183 bp of intronic sequences (represented by straight lines). Constructs harbored either the wild-type (WT) or one of two mutant alleles at the donor splice-site of intron 14: c.1495+2_1495+3insT and c.1495+1G>A. Also shown are the locations of primers used for RT–PCR analysis (indicated by arrows) and the obtained splicing products. Sizes of exonic and intronic fragments are indicated below them. **B**: WT and mutant constructs were transfected into Y79 cells, followed by RNA extraction and RT–PCR analysis. No PCR products were obtained from cells transfected with the empty pCMV-script vector. β-actin (*ACTB*), a 437 bp product, served as an internal control for RNA quality and quantity.

Due to the limited expression pattern of *TULP1* (mainly restricted to the retina [10]), we could not evaluate the effect of c.1495+2_1495+3insT on splicing in patient-derived RNA. Alternatively, we used an in vitro splicing assay approach. For this purpose, we created a set of minigene constructs harboring *TULP1* exons 12 to 15, flanked by 79–183 bp of intronic sequences, downstream of a CMV promoter. In addition to a WT construct, we generated constructs harboring two mutant alleles, both affecting the donor splice-site of intron 14: the c.1495+2_1495+3insT mutation identified in family TB13; and the c.1495+1G>A (IVS14+1G>A) mutation, which was previously identified in two Dominican kindreds [9] (Figure 4A). Constructs harboring either the WT or the mutant alleles were transfected into the Y79 retinoblastoma cell line, followed by RNA extraction and RT–PCR analysis. To analyze the results, we sequenced the splicing products derived from each construct. RNA derived from the WT construct was correctly spliced (a 430 bp product), while RNA derived from the mutant constructs yielded two types of aberrantly spliced products. In these two splicing products, an alternative acceptor splice-site, located 20 bp upstream of the original site within exon 15, was used. In one product (218 bp) exon 14 was skipped, while in another product (430 bp) a cryptic donor splice-site, located 20 bp downstream of the mutant splice-site within intron 14, was used (Figure 4).

The length of WT TULP1 protein is 542 amino acids. Skipping of exon 14 was expected to lead to premature translation termination, after the insertion of 37 incorrect amino acids, starting at position 442 of the protein. The use of a cryptic intron 14 donor splice-site (in combination with the alternative intron 15 acceptor splice-site) was expected to yield a protein with normal length (542 amino acids), however six amino acids (at positions 489–494 of the protein) were replaced.

## Discussion

The aim of the current study was to investigate the genetic basis for autosomal recessive severe early-onset RP in a consanguineous Israeli Muslim Arab family. Genetic analysis revealed a novel splice-site mutation of *TULP1*, c.1495+2_1495+3insT. We performed an in vitro splicing assay, which demonstrated that intron 14 donor splice-site harboring the c.1495+2_1495+3insT mutation is not efficiently recognized by the human splicing machinery. Although the exact effect of this splicing mutation on *TULP1* transcripts in vivo is not known, the expected outcome is incorrect splicing, leading to an abnormal protein product. Interestingly, two of the seven splice-site mutations of *TULP1* which have been reported to date, c.1495+1G>A (IVS14+1G>A) and c.1496–6C>A (IVS14–6C>A), affect the donor and acceptor sites of intron 14 [9] (Figure 4 and Table 1). This finding further demonstrates the importance of correct splicing of intron 14 for generation of normally functioning TULP1 protein.

The phenotype associated with *TULP1* mutations is usually described as LCA or early-onset RP (Table 1). In a recent paper, den Hollander et al. were able to predict the involvement of *TULP1* based on the disease phenotype, which they believed is specific for *TULP1.* This phenotype included early-onset nyctalopia with nystagmus; relative preservation of the isopters on kinetic perimetry, despite poor visual acuity; and a perifoveal yellow annular ring [16]. Clinical findings in affected individuals from family TB13, including early-onset of symptoms, nystagmus, myopia, central vision impairment, and pigmentary retinopathy, are consistent with this report (Figure 2). Interestingly, intrafamilial variability between affected individuals harboring the same *TULP1* mutation has been recently reported [20]. This report indicates that additional genetic as well as environmental factors contribute to the phenotypic outcome elicited by *TULP1* mutations.

In summary, *TULP1* is one of several genes known to cause early-onset autosomal recessive retinal degeneration. Some of these genes are major contributors to this phenotype. For example, over 85 pathogenic mutations have been reported for *CRB1*, over 55 for *GUCY2D*, and over 50 for *RPE65* (The Human Gene Mutation Database). In contrast, since the identification of the first *TULP1* pathogenic mutations in 1998 [9,11], only 22 distinct mutations of *TULP1* have been reported worldwide (Table 1). In a recent study, *TULP1* mutations were identified in approximately 4% of patients with LCA and juvenile RP [16]. This relatively small number suggests that *TULP1* mutations are a rare cause for early-onset retinal degeneration. Here we report a consanguineous Israeli Muslim Arab family segregating severe early-onset retinal degeneration due to a novel splice-site mutation of *TULP1*. This report expands the spectrum of pathogenic mutations of *TULP1*.
